# The Impact of Sexual Abuse in Patients Undergoing Colonoscopy

**DOI:** 10.1371/journal.pone.0085034

**Published:** 2014-01-15

**Authors:** Melianthe P. J. Nicolai, Josbert J. Keller, Lieke de Vries, Andrea E. van der Meulen-de Jong, Jan J. Nicolai, James C. H. Hardwick, Hein Putter, Rob C. M. Pelger, Henk W. Elzevier

**Affiliations:** 1 Department of Urology, Leiden University Medical Center, Leiden, the Netherlands; 2 Department of Gastroenterology, HAGA Teaching Hospital, The Hague, the Netherlands; 3 Department of Urology, Westeinde Hospital, The Hague, the Netherlands; 4 Department of Gastroenterology, Leiden University Medical Center, Leiden, the Netherlands; 5 Department of Medical Statistics, Leiden University Medical Center, Leiden, the Netherlands; Xi'an Jiaotong Univesity School of Medicine, China

## Abstract

**Background:**

Sexual abuse has been linked to strong effects on gastrointestinal health. Colonoscopy can provoke intense emotional reactions in patients with a sexual abuse history and may lead to avoidance of endoscopic procedures.

**Objective:**

To determine whether care around colonoscopy needs adjustment for patients with sexual abuse experience, thereby exploring targets for the improvement of care around colonoscopic procedures.

**Methods:**

Questionnaires were mailed to patients (n = 1419) from two centers within 11 months after colonoscopy. Differences in experience of the colonoscopy between patients with and without a sexual abuse history were assessed and patients' views regarding physicians' inquiry about sexual abuse and care around endoscopic procedures were obtained.

**Results:**

A total of 768 questionnaires were analyzed. The prevalence of sexual abuse was 3.9% in male and 9.5% in female patients. Patients born in a non-western country reported more sexual abuse (14.9%) than those born in a western country (6.3%; p = 0.008). Discomfort during colonoscopy was indicated on a scale from 0 to 10, mean distress score of patients with sexual abuse was 4.8(±3.47) compared to 3.5(±3.11) in patients without a sexual abuse history (p = 0.007). Abdominal pain was a predictor for higher distress during colonoscopy (β = −0.019 (SE = 0.008); p = 0.02, as well as the number of complaints indicated as reason for colonoscopy (β = 0.738 (SE = 0.276); p = 0.008). Of patients with sexual abuse experience, 53.8% believed gastroenterologists should ask about it, 43.4% said deeper sedation during colonoscopy would diminish the distress.

**Conclusions:**

Sexual abuse is prevalent in patients presenting for colonoscopy. Patients with a sexual abuse history experience more distress during the procedure and indicate that extra attention around and during colonoscopy may diminish this distress.

## Introduction

The prevalence of sexual abuse (SA) in modern western societies is estimated to be 12% to 25% for females and 8% to 10% for males [1]–[3]. SA has been linked to abdominal pain and functional gastrointestinal disorders [4], [5], more healthcare utilization and exacerbated (pelvic) pain perception [6]–[8]. In community samples, patients with an abuse history have a 1.5 to 2 times increased risk of reporting gastrointestinal (GI) complaints compared to non-abused individuals [9], [10]. Furthermore, SA has been linked to discomfort and traumatic reactions during pelvic examinations [11], [12] and has been reported to make patients feel vulnerable when undergoing invasive endoscopic procedures [13]–[15]. Disclosure of abuse in the gastroenterological setting may allow for earlier consultation with mental health professionals [6].

In a recent survey among gastroenterologists, the majority stated to be aware of the importance of inquiring about SA. Nevertheless many indicated a lack of training in dealing with abuse-related problems [16]. Patients' beliefs regarding routine direct inquiry about SA in gastroenterology practice have never been assessed and it is unknown whether colonoscopy is experienced differently by patients with a history of SA. Hypothesizing attention for SA in gastroenterology practice is limited; we were interested in patients' beliefs and attitudes regarding care for SA. We aimed to assess if patients with a SA history experience colonoscopic procedures differently and to identify whether care around colonoscopy needs adjustment for patients with SA experience, exploring targets for the improvement of patient-centered care around colonoscopic procedures.

## Materials and Methods

### Ethics statement

Written informed consent was obtained from all participants. The study was approved by the Medical Ethical Testing Committee of Southwest Holland.

### Patients and procedure

All patients ≥18 years old who had undergone colonoscopy in the selected timeframe were included. Patients were excluded if the procedure was performed under general anesthesia. Patients from the LUMC with inflammatory bowel disease (IBD) were excluded because participation in a study addressing sexuality in IBD was offered to those patients in the same period. IBD-patients from the HAGA teaching hospital were not excluded.

Within 11 months after the colonoscopy took place, the selected patients (n = 2348) received an introduction letter containing information about the study, a consent form and a freepost return envelope. Those returning the consent form with an affirmative answer received the questionnaire (in Dutch or English) within one month. The consent form contained an opt-out section in which reason for opting out was not asked for, as required by the medical ethical testing committee (MEC). One reminder letter was sent to non-respondents and one reminder letter was sent to respondents that agreed to participate but did not return a (completed) questionnaire.

### Colonoscopy

Colonoscopies were performed according to the routine protocols of both centers. Each endoscopy team consisted of an endoscopist (gastroenterologist, resident in gastroenterology or specialized nurse endoscopist) and one or more specialized endoscopy nurses. Conscious sedation with intravenous midazolam and fentanyl was used in all patients undergoing colonoscopy. Rarely, flumazenil or naloxone were given to counteract the sedative action of midazolam or fentanyl, and in some patients butylscopalamine was given, based upon the judgment of the performing endoscopist.

### Questionnaire

The questionnaire (appendix) was developed by the researchers. It was designed for both men and women. An expert panel with experience with the development of questionnaires checked its comprehensiveness and quality. A pilot study among 5 gastroenterologists, 3 residents in gastroenterology and 10 patients visiting one of the participating gastroenterology clinics was performed to assess the suitability, validity and comprehensiveness of the questionnaire.

The questionnaire included questions about sociodemographic data (age, gender, country of origin, way of referral and indication for colonoscopy), sexual function, micturition, SA history and the patient experience at colonoscopy. Furthermore, questions were included regarding patients' views on conversations about sexual function and SA with the gastroenterologist/physician. Several questions used were from The Pelvic Floor Inventories Leiden (PeLFIs), which is a validated tool to assess complaints of the pelvic floor and about SA [17], [18]. For respondents that confirmed a history of SA, additional questions were posed regarding desired forms of healthcare. No distinction was made between adult and childhood SA. Questionnaires were processed by independent researchers (M.P.N. and L. de V.), and could not be traced back to patient records. Data were strictly anonymous as prerequisite by the MEC. In order to process the data correctly the questionnaires were numbered and the corresponding address data were saved and handled separately. Because answering the questionnaire was potentially distressing for subjects, an independent sexologist/psychologist was available in case support was needed.

### Statistical analysis

Results were summarized by reporting responses on all surveyed items. Frequencies of demographic characteristics and answers to the questions were all presented. Numerical demographic values were summarized as mean (SD). Differences in numerical data between demographic groups were analyzed with independent sample t-tests. χ^2^ Tests were used to assess association between categorical respondents' characteristics and categorical responses. Linear regression analysis was used to identify predictors of distress caused by colonoscopy and to correct for these factors. Statistical significance was defined as p<0.05, all tests were two-sided. Confidence intervals were defined as 95%. Analyses were conducted using SPSS release 20 (SPSS Inc., Chicago, IL, USA), GraphPath Prism 5 was used to design the figures.

## Results

### Subjects

**Figure 1:**
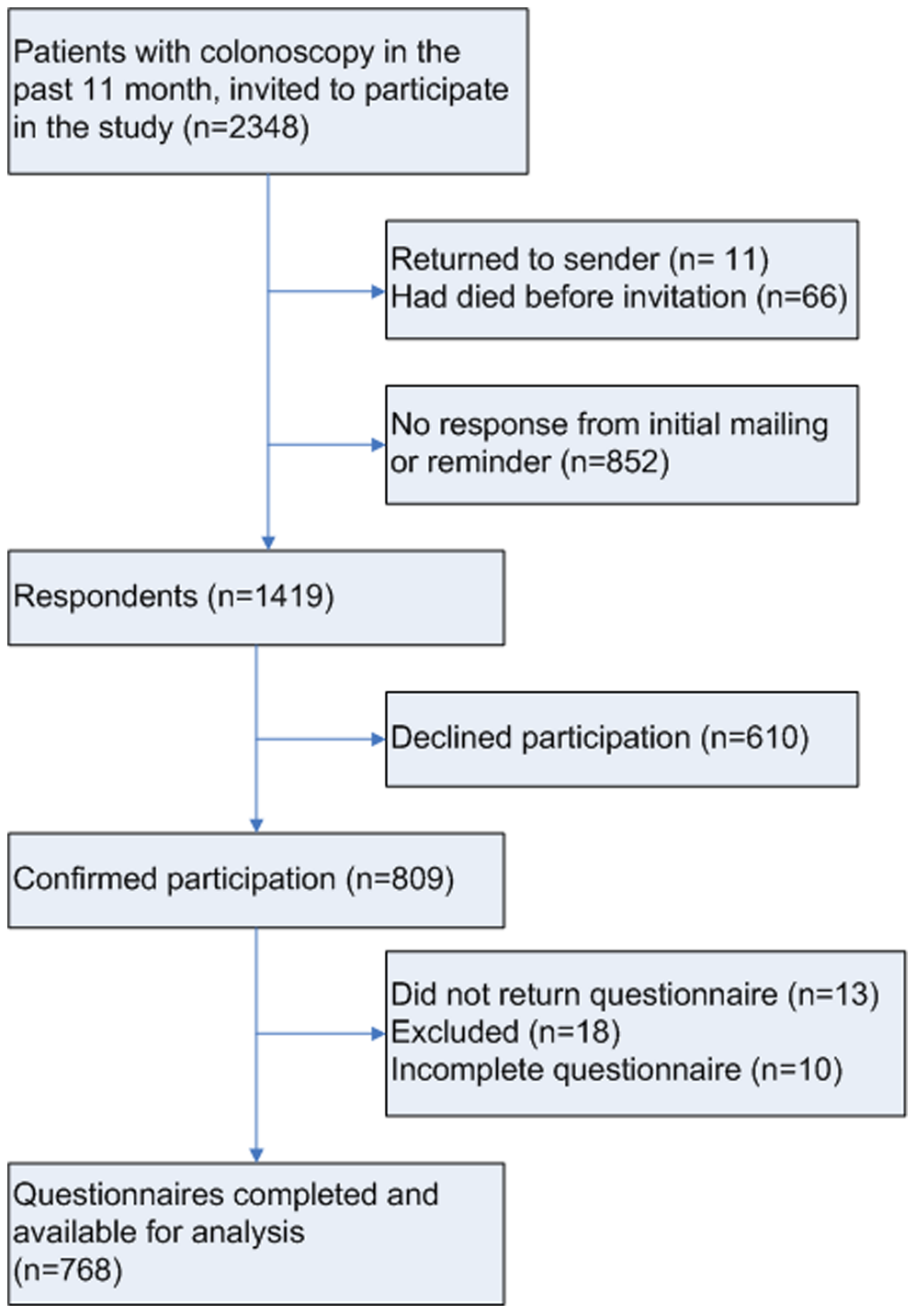
Study flow diagram.

From the 2348 patients who received the information letter and consent form, 1419 forms were returned (60.4%). Of these respondents, 610 (43.1%) declined participation and 809 (57.0%) were willing to participate. Of the 809 patients that received the questionnaire, 13 did not return it and 10 were incomplete for more than 30% and therefore excluded. One respondent was excluded because she indicated not to remember anything about the colonoscopy. Seventeen respondents were excluded because they underwent sigmoidoscopy and should not have been invited in the first place. The above led to a total of 768 questionnaires available for analysis (Figure 1). Mean time span between colonoscopy and return of the competed questionnaires was 274.0 days (±70.3).

Mean age of participants was 61.2 years (±14.5 years), female participants were younger than male participants with a mean age of 59.9 (±15.3 years) compared to 62.8 (±13.2) years respectively (p = 0.02). There was no difference in age between patients that declined participation (mean age 61.4±15.9) and participants. Non-respondents were younger than participants with a mean age of 53.8 years (±16.4) (p<0.001). Of the included patients, 43.9% were male. The majority of respondents were born in the Netherlands (88.4%, n = 674), 3.4% was from an other western country (n = 26) and 8.2% from a non-western country (including Turkey, Morocco, Surinam, and the Dutch Antilles, n = 63) (Table 1). From the respondents included for analysis, 81.4% underwent colonoscopy in the general teaching hospital and 18.4% in the tertiary referral center (LUMC).

**Table 1:** Characteristics of study sample.

Gender n(%)	Female: 429 (56.1)^a^	Male: 336 (43.9)^a^	Difference *p*-value
Age, mean (SD), years	59.9 (15.3)	62.8(13.2)	0.02
Age, ≤39 years, n(%)	41 (9.6)	25 (7.4)	0.08
Age, 40–49 years, n(%)	63 (14.7)	25 (7.4)	0.19
Age, 50–59 years, n(%)	84 (19.6)	60 (17.9)	0.07
Age, 60–69 years, n(%)	127 (29.6)	124 (36.9)	0.04
Age, 70–79 years, n(%)	74 (17.2)	72 (21.4)	0.34
Age, 80–89 years, n(%)	38 (8.9)	29 (8.6)	0.004
Age ≥90 years, n(%)	2 (0.5)	1 (0.3)	n/a

Country of origin n (%)	Female n = 428^a^	Male n = 335^a^	Difference *p*-value
The Netherlands	378 (88.3)	296 (88.4)	0.98
Other West-European country	13 (3.0)	9 (2.7)	0.78
Morocco	2 (0.5)	2 (0.6)	0.80
Turkey	3 (0.7)	4 (1.2)	0.48
Surinam	12 (2.8)	10 (3.0)	0.89
Dutch Antilles	3 (0.7)	4 (1.2)	0.48
Elsewhere western	2 (0.5)	2 (0.6)	0.80
Elsewhere non-western	15 (3.5)	8 (2.4)	0.38

Reason for colonoscopy^b^ n (%)	Female n = 428^b^	Male n = 336^b^	Difference *p*-value
Abdominal pain	150 (35.0)	81 (24.1)	0.001
Rectal blood loss	110 (25.6)	108 (32.1)	0.05
Surveillance (hereditary) colon carcinoma/polyposis/polyps^c^	111 (25.9)	97 (28.9)	0.37
Changes defecation pattern	104 (24.3)	68 (20.2)	0.18
Chronic diarrhea, constipation or mucus in stool	37 (8.6)	15 (4.5)	0.02
Inflammatory Bowel Disease	18 (4.2)	22 (6.5)	0.15
Pain anal region	18 (4.2)	20 (6.0)	0.27
Anemia	9 (2.1)	14 (4.2)	0.10
Other^d^	38 (8.9)	31 (9.2)	0.51

n/a =  not applicable.

a. Based on data from n = 768 respondents of which 429 women and 336 men (due to missing values), columns do not necessarily add to 768.

b. Multiple answers were possible.

c. Significantly more patients with the indication ‘surveillance for colonoscopy for hereditary colon carcinoma/polyposis/polyps’ were seen in the tertiary center (41.3%) compared to the general teaching hospital (23.6%; p<0.001).

d. Under which: loss of weight, diarrhea, eating disorders etc.

Indications for colonoscopy were listed in Table 1. The most common indications were abdominal pain (not specified) (30.4%) and rectal blood loss (28.4%).

Almost three quarter of respondents indicated one complaint to be the reason for colonoscopy (73.3%; n = 563), 20.2% (n = 155) indicated two complaints, 5.2% (n = 40) three and 1.0% (n = 8) indicated four different reasons for the colonoscopy.

The majority was referred for colonoscopy by the general practitioner (48.8%). Almost a third (27.3%) was indicated for colonoscopy by the gastroenterologist him/herself and 11.1% by a physician in internal medicine (otherwise), 3.7% was referred by a surgeon.

Voiding complaints were present in 33.1% of respondents (n = 254): 52.5% of them mentioned frequency, 27.5% urgency and 24.3% urinary incontinence.

A quarter of the patients (25.7%) reported sexual dysfunction (n = 197), this was more prevalent in male (34.5%) than in female patients (18.4%).

### Sexual abuse

**Figure 2:**
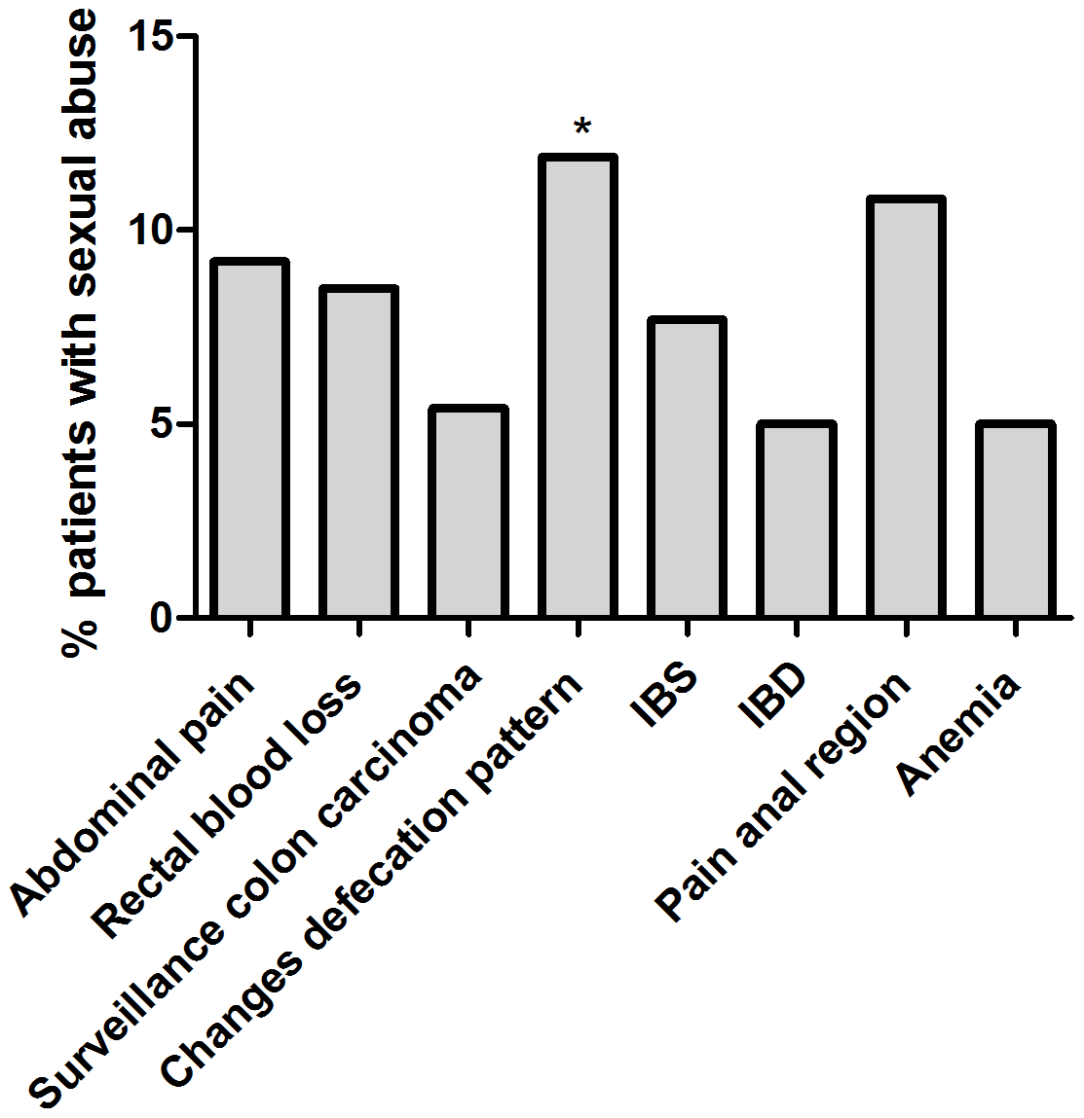
Indication for colonoscopy in patients with sexual abuse. * p = 0.006. Based on results for 53 patients, multiple answers were possible

SA was reported by 53 (7.0%) of the 752 respondents that answered the question ‘have you ever been a victim of sexual abuse?’ Sexual abuse occurred in 40 (9.5%) of females (n = 421) and in 13 (3.9%) of males (n = 331). Patients born in a non-western country reported more SA compared to patients born in the Netherlands or another western country (14.9% versus 6.3%; p = 0.008). More details about the distribution of sexual abuse can be found in Table 2 and Figure 2.

**Table 2:** Distribution of sexual abuse prevalence.

Patients Characteristics	Female n(%)	Male n(%)	Difference *p*-value
**Total sexual abuse: 53 (7.0)**	**40 (9.5)**	**13 (3.9)**	**0.003**
**Age**			
≤39 years	9 (22.5)	1 (1.5)	0.05
40–49 years	9 (22.5)	2 (2.3)	0.42
50–59 years	7 (17.5)	0	0.02
60–69 years	9 (22.5)	4 (1.6)	0.18
70–79 years	3 (7.5)	5 (3.5)	0.45
80–89 years	3 (7.5)	1 (1.6)	0.39
Age ≥90 years	0	0	n/a

Country of origin	Female n(%)^a^	Male n(%)^a^	Difference *p*-value
The Netherlands	32 (80)	10 (77.0)	0.93
Other West-European country	1 (2.5)	0	0.57
Turkey	0	0	n/a
Morocco	0	0	n/a
Surinam	2 (5.0)	0	0.42
Dutch Antilles	0	2 (15.4)	0.019
Elsewhere western	1 (2.5)	0	0.57
Elsewhere non-western^c^	4 (10.0)	1 (7.7)	0.82
Total Western country	33 (82.5)	10 (76.9)	0.004
Total Non-Western country^ d^	7 (17.5)	3 (23.1)	0.412

Based on data from n = 752 respondents of which 421 women and 331 men (due to missing values).

Percentage of the total respondents with sexual abuse, by gender.

Total exceeds 100% because multiple answers where possible.

Namely: Brazil, Colombia, Egypt (male victim), Indonesia, Iran and Russia.

Included: Morocco, Surinam, Dutch Antilles, elsewhere non-western.

Thirteen of 722 respondents indicated that a physician inquired about SA (1.8%), six were asked by the GP (0.8%) and three by the gastroenterologist (0.4%). Of the patients that reported SA (n = 53), 24.5% had professional help to deal with the experience and 64.1% said to have found a way to cope with it. More than half of patients with SA believed the gastroenterologist should ask about it (53.8%). Of them (n = 28), 35.7% said they would benefit from advice about dealing with the past, this option was marked significantly more often in male (80.0%) than in female patients (26.0%; p = 0.023). To the question “Do you think gastroenterologists need to have more training on the subject sexual health?” 46.7% said ‘yes’, 25.1% said ‘no’ and the remaining 21.6% answered with “I do not know”.

Of patients with SA, 24 (45.3%) indicated gastroenterologists should not ask about SA (n = 24). One of the most common reasons for this answer was: “I am unable to talk about it”. Female patients gave this answer significantly more often than male patients (29.2% vs. 0% respectively, p = 0.05), see Table 3.

**Table 3:** Should gastroenterologists ask about sexual abuse? **Answered by patients with a sexual abuse history.**

**“No, the GE should not ask about SA”, n(%)**	**24 (45.3)^a^**
**If not, what is the reason you do not want to talk about it?^b^**	
I am ashamed of it	6 (25.0)
I do not believe the GE can help me with this problem	7 (29.1)
I am not able to talk about it	7 (29.1)
I am afraid to tell	5 (20.8)
It is not important for me anymore	7 (29.1)
It is too intimate to discuss	6 (25.0)
**“Yes, the GE should ask about SA”, n(%)**	**28 (52.8)^a^**
**If yes, ‘what should the GE do after you told him about the SA?’^b^**	
Just listen to me	7 (25.0)
Give me some advise about dealing with it	10 (35.7)
Refer me to psychologist	6 (21.4)
Refer me to a sexologist	6 (21.4)
Refer me to a pelvic floor physiotherapist	3 (10.7)
Refrain from performing a colonoscopy	2 (7.1)
Give me some information to read about it	10 (35.7)

GE =  gastroenterologist, SA =  sexual abuse.

a. Columns do not add to 53 because one patient with SA did not answer these questions.

b. Multiple answers were possible.

Patients without SA experience (n = 715) were asked to answer the question: “Do you think gastroenterologists should ask their patients about sexual abuse?” 36% said “Yes” and 64% said “No”. However, of all respondents (n = 768), only 23.7% believed a question about SA in an intake questionnaire would be peculiar (n = 182). No significant difference was seen between patients with and without a SA history concerning this question (22.4% resp. 24.7%; p = 0.723). Of the respondents stating that a question about SA in an intake questionnaire would be peculiar, the reason for this answer was mostly because they did not see the relationship (56.0%) or the relevance (31.1%) of SA in the context. No significant differences were seen between male and female respondents concerning their answers to the above questions.

Victims of SA noted significantly more sexual dysfunction (64.0%) compared to those without SA (24.4%; p<0.001), more micturition complaints (58.8% versus 30.9%; p<0.001) and a combination of both complaints (37.3 versus 11.4%; p<0.001).

Significantly more patients with SA history indicated a change in bowel habit as the reason for colonoscopy (p = 0.006) (Figure 2). Abdominal pain was significantly correlated to sexual abuse in men (r = 0.572, p = 0.031) and to a lesser extent in women (r = 0.291, p = 0.052). More than one GI-complaint was correlated with SA (r = 0.1, p = 0.006), as well as age under 60 years (r = −0.108, p = 0,003).

### Discomfort during colonoscopy

**Figure 3:**
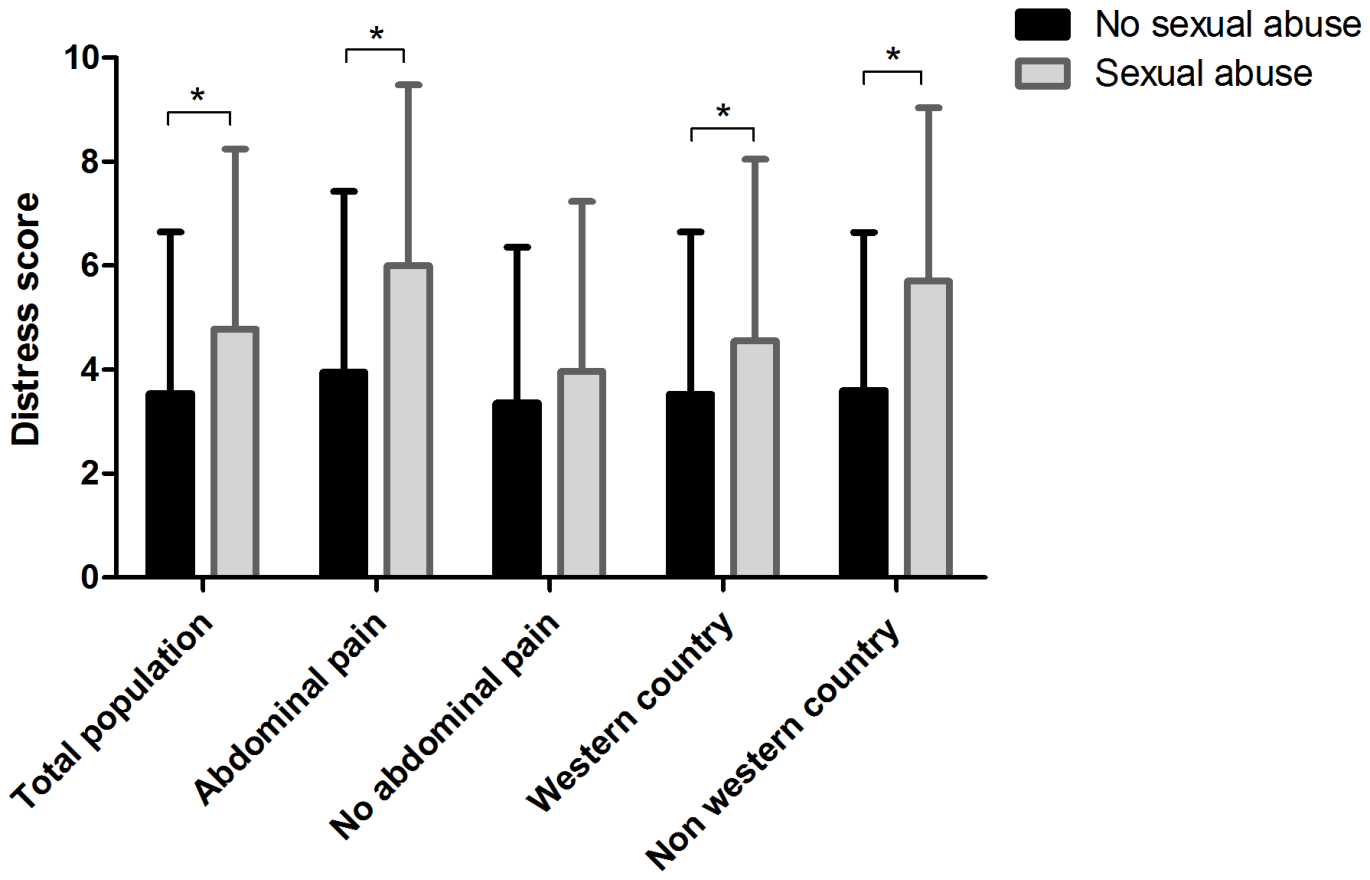
Distress during colonoscopy in patient with and without sexual abuse. * = significant difference.

Patients were asked to rate discomfort experienced during their last colonoscopy on a 10-point Likert scale in which 0 meant “no discomfort” and 10 meant “extreme discomfort”. Patients with a sexual abuse history rated more discomfort (mean score 4.78±3.47) compared to non-abused subjects (mean score 3.54±3.11; p = 0.007) (Figure 3). Using linear regression, we controlled for age, gender, ethnicity, indication(s) for colonoscopy, and time between the colonoscopy and filling in the questionnaire. Several factors were found to influence the distress. Age and abdominal pain were influencing factors (β = −0.019 (SE = 0.008); p = 0.02 respectively β = 0.354 (SE = 0.156); p = 0.024) as well as the number of complaints presented (β = 0.738 (SE = 0.276); p = 0.008) and country of origin (Table 4). After controlling for these factor, sexual abuse was still a significant predictor for distress during colonoscopy (β = 0.991 (SE = 0.466); p = 0.034). Time between participation in the study and colonoscopy was not of influence (β = <0.001 (SE = 0.022); p = 0.914), see Table 4.

**Table 4:** Distress experienced during colonoscopy.

Sexual abuse			No sexual abuse		
	Mean (±SD)	Total (n)	Mean (±SD)	Total (n)	*p*-value
**Total**	4.8(±3.47)	50^a^	3.5(±3.11)	684	0.007
**Male**	5.0(±3.82)	11	3.2(±2.93)	311	0.052
**Female**	4.7(±3.47)	39	3.8(±3.23)	371	0.102
**Age≤60 years**	4.8(±3.55)	32	3.8(±3.06)	288	0.082
**Age> 60years**	4.7(±3.41)	18	3.4(±3.13)	396	0.071
**With abdominal pain**	6.0(±3.48)	20	4.0(±3.48)	204	0.009
**No abdominal pain**	4.0(±3.26)	30	3.4 (±2.99)	480	0.289
**Western country**	4.6(±3.50)	40	3.5 (±3.12)	631	0.048
**Non-western country**	5.7(±3.34)	10	3.6(±3.03)	53	0.053
**Sexual complaints**	5.3(±3.38)	31	3.7(±3.18)	153	0.011

Of the respondents with SA three did not fill in the distress-score.

Based on answers of 734 respondents, 34 respondents did not indicate distress during colonoscopy.

**Figure 4:**
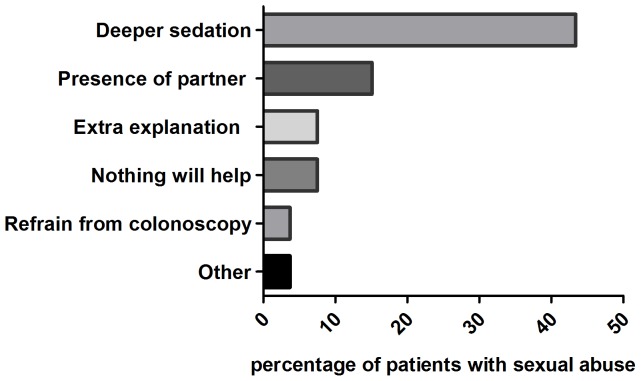
Possible options to diminish distress during and around colonoscopy, answers of patients with a history of sexual abuse^a^. a. Answers to the multiple choice/open question: “The colonoscopy experience would have been easier/more comfortable for me if…” Based on answers of 51 patients with sexual abuse experience. b. In the free space provided, one patient said: only start about sexual abuse if it has to do with the complaints, and one said ‘not declare you as depressed’.

Patients with SA were asked to indicate which changes around and during colonoscopy would have made the procedure easier for them. Of 51 patients with a sexual abuse history that answered this question, a quarter (25.4%, n = 13) said the procedure went well, however 88.3% (n = 45) identified one or multiple options that would make the endoscopic procedure less uncomfortable, these options can be found in Figure 4.

## Discussion

This cohort showed a SA prevalence of 3.9% in men and 9.5% in women. Many of the patients which experienced SA were born in a non-western country. Importantly, a SA history was associated with more discomfort during the colonoscopic procedure. Abdominal pain, multiple gastrointestinal complaints at presentation for colonoscopy and sexual dysfunction were associated with SA and more discomfort during the procedure. Most sexually abused patients indicated that gastroenterologists should ask about it before performing colonoscopy. Several minor adjustments where indicated as options to diminish distress during and beforehand of the procedure.

The prevalence rates of SA in our population were comparable with those obtained in a survey among patients visiting a general urology practice in the Netherlands [19]. In studies assessing SA in selected samples of patients with (functional) gastrointestinal illness from referral gastroenterology clinics, up to 44% of women and 11% of men reported sexual abuse [20]–[23]. However, when the same questions about sexual abuse were posed in community samples, the reported prevalence rates of SA with physical contact (touching and penetration) were comparable to the rates found in present study [24], [25]. Because we were restricted by the MEC the specific form of abuse experience could not be asked about, therefore the demarcated question: ‘Have you ever been a victim of sexual abuse?’ was used. Because this question implicates sexual abuse wíth contact, patients with less explicit forms of abuse such as exposure to exhibitionists or (verbal) sexual intimidation will not have felt addressed. This will have led to an underestimation of sexual abuse in our sample, together with the effects of selection and non response bias.

To minimize potential biases we provided detailed assurances of confidentiality, pilot tested and refined the instrument and used balanced keying. Still, traumatized patients may have elected not to participate in the study to avoid reliving painful memories and nonrespondents may have had no affinity with the subject or may have considered their participation irrelevant for the study. Some patients with SA may have refused colonoscopy and therefore did not receive the invitation to participate in the study. The initial response rate was no higher than 32.7% (768/2348), but in the first mailing (2348 patients) only the letter with study information was sent out. Patients were requested to send back the consent form in order to obtain a questionnaire or opt out of the study. This step must therefore be regarded as study recruitment; a part of this initial sample was not eligible due to changes in address, illness, memory loss or death. Therefore the eligible response rate of 54.1% (768/1419 respondents) should be used and considered representative [26].

An obvious limitation regarding the interpretation of discomfort experienced during colonoscopy is the absence of control for confounding variables such as length of the procedure and the amount of additional sedation used. Due to restrictions imposed by the MEC, we could not link questionnaires with patient files and therefore were unable to obtain these data.

Furthermore, recall bias may have occurred. The validity of retrospective reports by adults of their own adverse experiences in childhood has been extensively studied; so called infantile amnesia, the effect of mood and false or recovered memory may all influence retrospective recall of traumatic experiences [27], [28]. In response, methods for addressing the issues of reporting unreliability and recall bias in retrospective reports of child sexual abuse were explored. The influence of recall bias was found to be small, accounting for less than 1% of the reporting variance [29]. Moreover, longitudinal data have to rely on retrospective recall for measures of experiences since the last interview as well, which will often involve reporting over a period of several years. And because longitudinal data are very expensive to collect, the discussion about methodology ended with the conclusion that retrospective reports about childhood abuse have a worthwhile place in research until better methods are found [30].

In spite of these limitations, this was the first study obtaining an inventory of SA in colonoscopy patients, identifying their needs regarding the colonoscopic procedure and comparing experienced discomfort during colonoscopy between patients with and without SA. Our results were consistent with prior research showing that female patients with a history of SA reported more discomfort and anxiety during gynecological examination [12], [31]–[33]. And confirmed the link between sexual abuse, abdominal pain and multiple GI-complaints already found in the early nineties by Drossman et al. which has been verified in many studies afterwards [6], [34], [35].

The reason a history of SA results in more pain during pelvic examinations may be explained by a variety of neural and humoral pathways that link brain, pelvic floor and gut [36]. Alterations in psychopathological and cortico-limbic pain modulatory systems have been described as mediating mechanisms for the association between SA and gastrointestinal disorders [7]. In addition, anxiety and trauma, especially SA, are significantly associated with dysfunction of the pelvic floor [37], leading to FGID [37], [38], dyspareunia [39], dysfunctional voiding [40] and chronic pelvic pain [41]. In patients with IBS and a history of SA significantly more pain was reported to aversive rectal distention (similar to colonoscopy) compared with patients with IBS or abuse alone. Patients with IBS and SA reported higher pain scores and greater anterior mid cingulate activation with rectal distention than patients with either IBS or SA [42]. It is therefore remarkable that, however significant, the differences in mean distress scores found between patients with and without SA where rather mild (3.5 versus 4.8). This could be due to the fact that anxiety, depression and post-traumatic stress disorders play a major part in both perception of pain and symptoms as well [43]. A recent study using in-person interviews among gastroenterologists and endoscopy nurses to obtain information regarding development of psychological and/or physical symptoms after gastrointestinal endoscopy, fourteen of the 29 gastroenterologists (48%, 95% CI  = 30.7–66.2%) reported encountering patients with new onset psychological symptoms lasting for more than a month after upper endoscopy or colonoscopy. A history of psychiatric illness was noted in 11 of 19 patients (58%) and a history of sexual abuse was noted in five of 19 patients (26%). Physicians reported that the endoscopic procedure was longer or more difficult than usual in six of 19 patients and that four of 19 patients had requested to prematurely terminate the procedure [44]. Our study specifically focused on SA in relation to distress experienced during colonoscopy. Patients with psychological problems, but without a SA history may have caused the diluted difference in discomfort between patients with and without SA.

Given the mounting evidence of the long-term detrimental effects of SA, especially as regards to gastrointestinal complaints [37], routine inquiry about SA in the gastroenterology practice might be expected. However, only 1.8% of the responding patients reported their gastroenterologist asked about ‘negative sexual experiences’ or abuse during consultation. Accordingly, a recent study among gastroenterologists showed that 2.5% of them asked female patients and 0.6% asked male patients about SA before performing colonoscopy. However, the majority of gastroenterologists' rated it as important to pay more attention to SA during their training [16].

From a patients' perspective, in the present study we found that three-quarters of the patients would not find it peculiar if a question about SA would be asked in an intake questionnaire beforehand of the colonoscopy. On the contrary, a third of respondents without SA believed the gastroenterologist should ask about it, and of the patients with SA experience, more than half believed so. More inquiry about SA may result in a better understanding between physician and patient, which is known to be the most important aspect of the pelvic examination experience for women [45]. If the endoscopist is informed about patients' traumas, compassionate and individualized care around and during the endoscopic procedure might diminish its impact. This study indicated that offering options such as use of a chaperone, deeper sedation and/or clear communication about the steps taken during the procedure may decrease discomfort, helping the patients to undergo the examination. Offering extra attention to patients with a history of sexual abuse may limit avoidance of transanal endoscopic procedures and may improve cooperation during procedures and therapy.

In conclusion, the results of this study have several implications for clinical practice. At least a tenth of women and up to four percent of men undergoing colonoscopy indicated experiencing SA. Colonoscopy was shown to be more distressing for patients with a history of SA. Consequently, especially in the gastroenterology practice this subject deserves attention. Discussing SA in medical practice seems difficult for both physician and patient. This study indicates that the use of a standardized intake questionnaire including questions about SA could be a solution to this problem.
